# Supplementary material for: Transcription factor EB regulates phosphatidylinositol-3-phosphate levels that control lysosome positioning in the bladder cancer model
Source: Commun Biol. 2023 Jan 28;6:114. doi: 10.1038/s42003-023-04501-1 (PMC9884284; doi:10.1038/s42003-023-04501-1)
Supplement: Supplementary file 2 — Supplementary Figures [file 42003_2023_4501_MOESM2_ESM.pdf]

Transcription Factor EB regulates phosphatidylinositol-3-phosphate levels that control lysosome positioning in the bladder cancer model

Pallavi Mathur[1,2,3], Camilla De Barros Santos[1,2], Hugo Lachuer[1,2,3], Julie Patat[3,4], Bruno Latgé[1,2], François Radvanyi[1,2], Bruno Goud[1,2], Kristine Schauer[1,2,3,4*]

[1]Centre National de la Recherche Scientifique, UMR144, 75005 Paris, France
[2]Institut Curie, PSL Research University, 75248 Paris, France
[3]Institut Gustave Roussy, INSERM UMR1279, 94805 Villejuif, France
[4] Paris-Saclay University, 91190 Gif-sur-Yvette, France

Supplementary Figures

Figure S1:

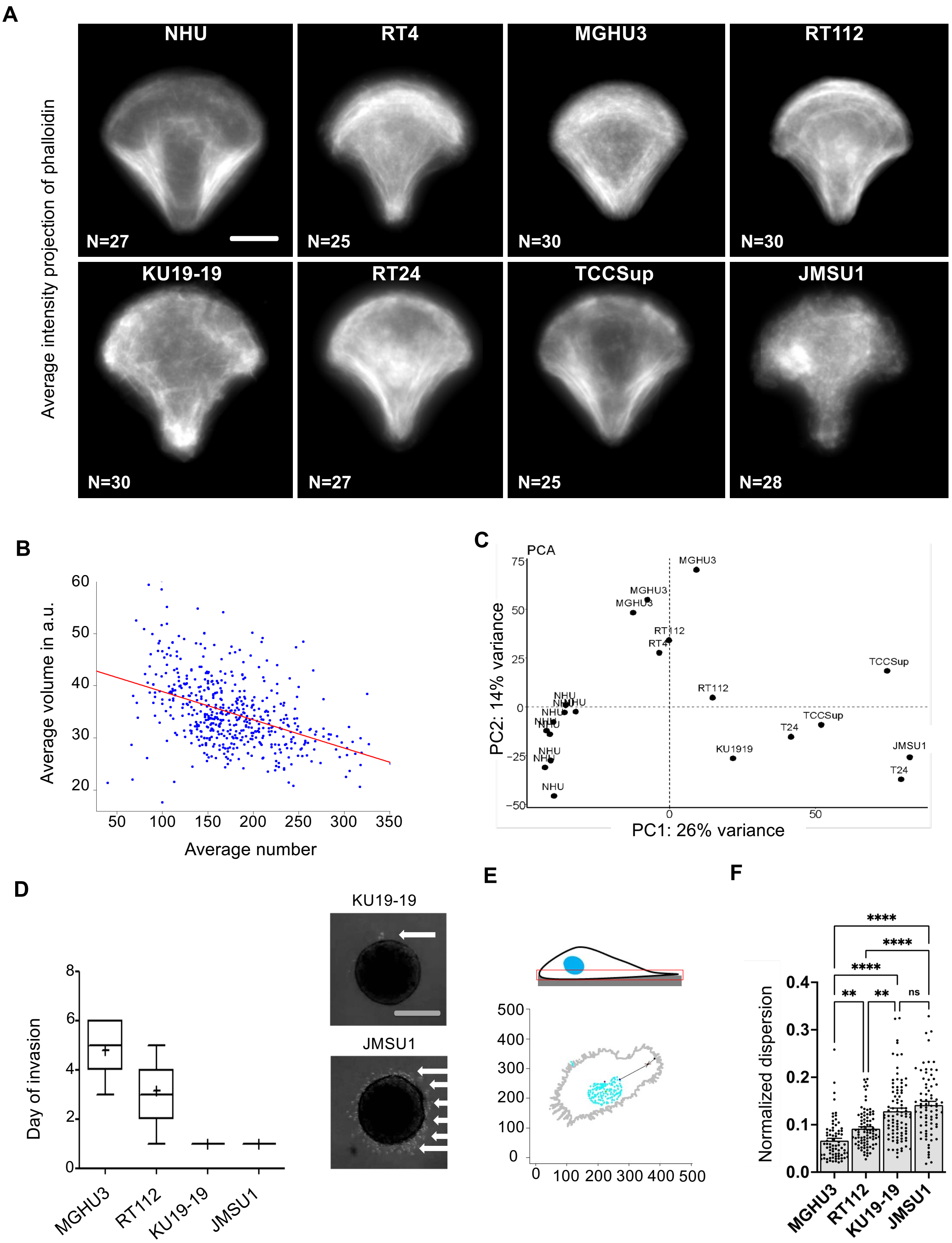

Figure S1. Cell lines representing high-grade bladder cancers are characterized by a peripheral positioning of lysosomes

A. Average intensity projections of the actin cytoskeleton visualized by phalloidin of n cells of normal human urothelium (NHU) and bladder cancer cell lines RT4 (ATCC® HTB-2™), MGHU3 (Lin et al., 1985), RT112 (Marshall et al., 1977), KU19-19 (Tachibana et al., 1995), T24, TCCSup (Nayak et al., 1977), JMSU1 (Morita et al., 1995). Scale bar equals 10 µm. **B**. Correlation analysis between average endolysosomal volume and average numbers per cell shows a weak (R^2=0.19) but significant association; p-value < 0.001 in a t-test for correlation. **C**. Principal component analysis of transcriptome data of normal human urothelium (NHU) cells and the bladder cancer cell lines RT4, MGHU3, RT112, KU19-19, T24, TCCSup and JMSU1. **D**. Average day of invasion from spheroids into collagen matrix of MGHU3 (n=13), RT112 (n=9), KU19-19 (n=5), and JMSU1 (n=8), and representative images of 3D spheroids from KU19-19 (upper panel) and JMSU1 (lower panel) at 1 day after matrix embedding. White arrow indicates invasion of collagen matrix by escaping cells. Scale bar equals 500 µm. **E**. Schematic representation of the analysis of endolysosome distribution in classical cell culture conditions (see **F**). **F**. Normalized lysosome dispersion in non patterned MGHU3, RT112, KU19-19 and JMSU1 cells based on statistical inertia measurement (=averaged squared distance to the center of mass normalized to cell size) for n>60 cells per cell line analyzed, **** p < 0.0001 in a Kruskal-Wallis test with Dunn's test for multiple comparison.

Figure S2:

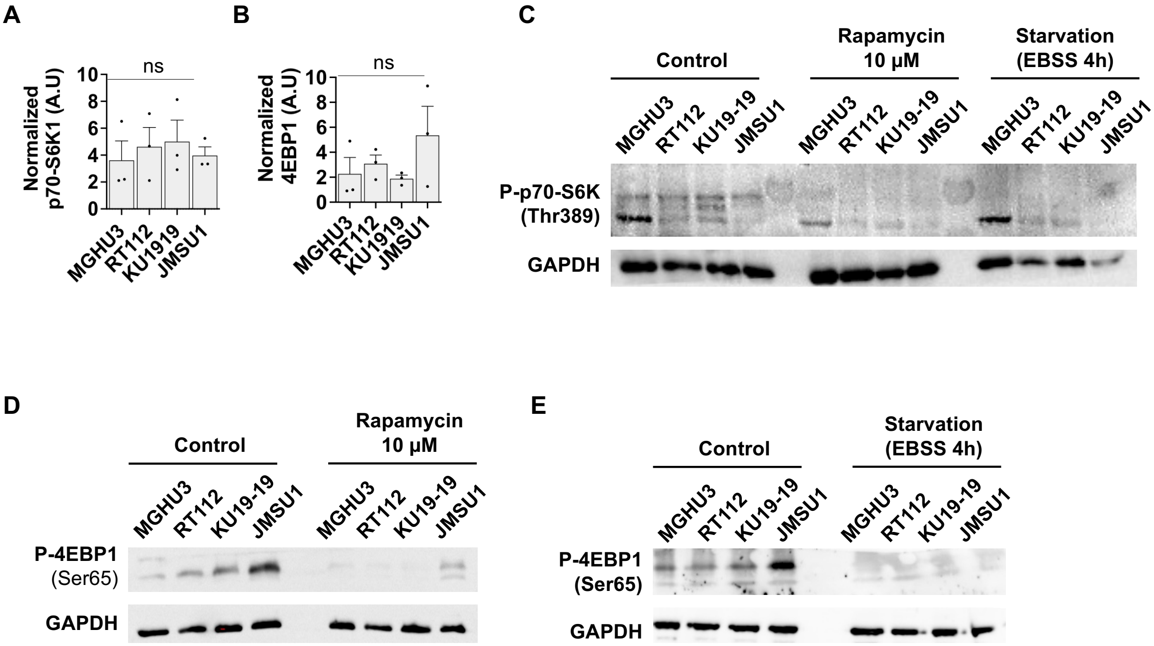

Figure S2. Dispersed lysosomes reveal alterations in mTORC1 signaling

A. Quantification of total p70-S6 Kinase 1 levels from n=3 Western Blot experiments in MGHU3, RT112, KU19-19 and JMSU1 (see also Figure 2C). ns $p>0.05$ in a Kruskal-Wallis test with Dunn's test for multiple comparison. Error bars show SEM. **B**. Quantification of total 4EBP1 levels from n=3 Western Blot experiments in MGHU3, RT112, KU19-19 and JMSU1 (see also Figure 2D). ns $p>0.05$ in a Kruskal-Wallis test with Dunn's test for multiple comparison. Error bars show SEM. **C**. Western Blot analysis of phosphorylated p70-S6 Kinase 1 (P-p70-S6K1 Thr389) and GAPDH loading control in MGHU3, RT112, KU19-19 and JMSU1 cells in control conditions (full media) and after treatment with rapamycin at 10 µM for 2 h or grown under starvation in EBSS (Earle's Balanced Saline Solution) for 4 h. **D.** Western Blot analysis of phosphorylated 4EBP1 (P-4EBP1 Ser65) and GAPDH loading control in MGHU3, RT112, KU19-19 and JMSU1 cells in control conditions (full media) and after treatment with rapamycin at 10 µM for 2 h. **E**. Western Blot analysis of phosphorylated 4EBP1 (P-4EBP1 Ser65) and GAPDH loading control in MGHU3, RT112, KU19-19 and JMSU1 cells in control conditions (full media) and grown under starvation in EBSS (Earle's Balanced Saline Solution) for 4 h.

Figure S3:

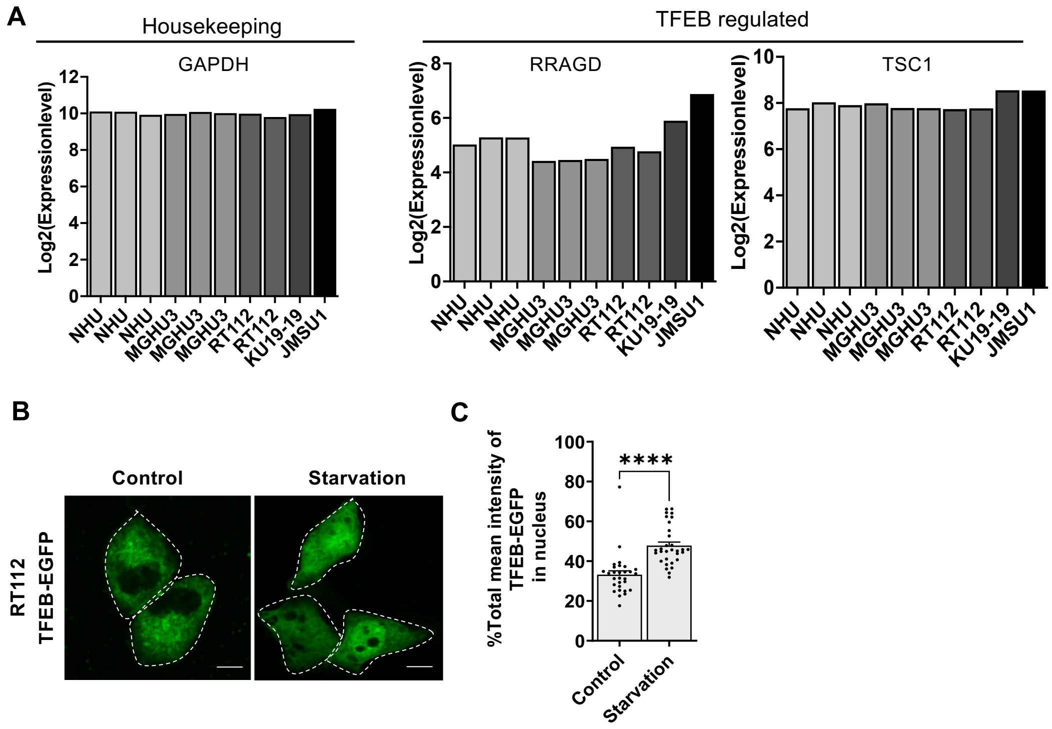

Figure S3. TFEB regulation in bladder cancer cell lines

A. Normalized Log2 RNA expression levels of housekeeping gene (GAPDH) and TFEB regulated genes (*RRAGD, TSC1*) in NHU, MGHU3, RT112, KU19-19 and JMSU1. **B**. Representative images of RT112 cells transfected with TFEB-EGFP for 72 h in control conditions (full media) and grown under starvation in EBSS (Earle's Balanced Saline Solution) for 4h. Scale bars are 10 µm. **C**. Quantification of the nuclear fraction of the total mean TFEB-EGFP fluorescent intensity in control (full media) and starvation (EBSS, 4h) treated JMSU1 cells (for n>15 cells in each condition). **** p >0.0001; Mann-Whitney test. Error bars are SEM.

Figure S4:

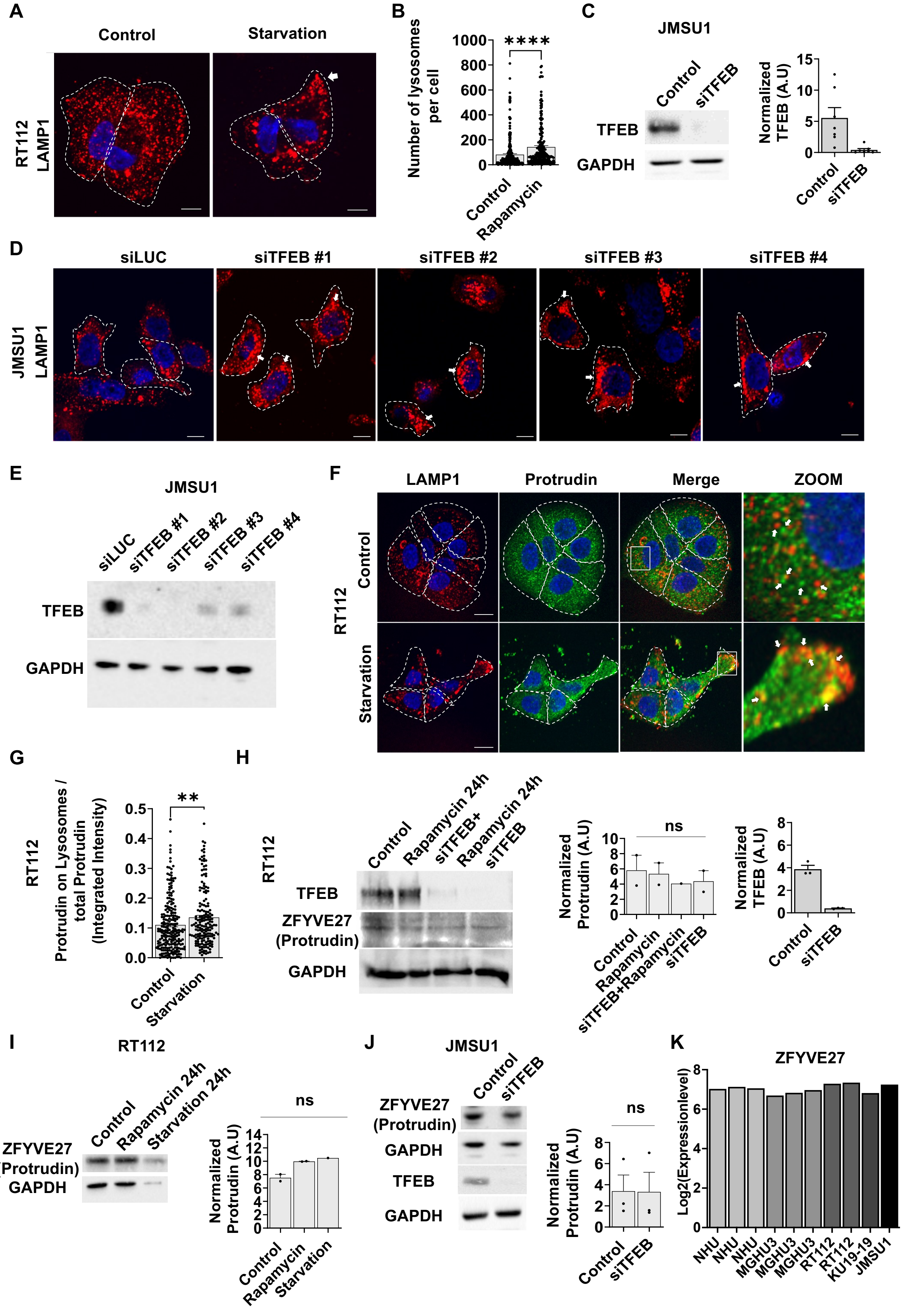
A
Control
Starvation
RT112
LAMP1
B
Number of lysosomes per cell
****
Control
Rapamycin
C
JMSU1
Control
siTFEB
TFEB
GAPDH
Normalized TFEB (A.U)
D
siLUC
siTFEB #1
siTFEB #2
siTFEB #3
siTFEB #4
JMSU1
LAMP1
E
JMSU1
siLUC
siTFEB #1
siTFEB #2
siTFEB #3
siTFEB #4
TFEB
GAPDH
F
LAMP1
Protrudin
Merge
ZOOM
RT112
Control
Starvation
G
RT112
Protrudin on Lysosomes / total Protrudin (Integrated Intensity)
**
Control
Starvation
H
RT112
Control
Rapamycin 24h
siTFEB+ Rapamycin 24h
siTFEB
TFEB
ZFYVE27 (Protrudin)
GAPDH
Normalized Protrudin (A.U)
ns
Control
Rapamycin
siTFEB+Rapamycin
siTFEB
Normalized TFEB (A.U)
Control
siTFEB
I
RT112
Control
Rapamycin 24h
Starvation 24h
ZFYVE27 (Protrudin)
GAPDH
ns
Normalized Protrudin (A.U)
Control
Rapamycin
Starvation
J
JMSU1
Control
siTFEB
ZFYVE27 (Protrudin)
GAPDH
TFEB
GAPDH
ns
Normalized Protrudin (A.U)
Control
siTFEB
K
ZFYVE27
Log2(Expressionlevel)
NHU
NHU
NHU
MGHU3
MGHU3
MGHU3
RT112
RT112
KU19-19
JMSU1

Figure S4. Lysosome positioning correlates with TFEB activation in bladder cancer cells

A. Immunofluorescence staining of the lysosomal-associated membrane protein 1 (LAMP1, CD107a) in control (full media) and starvation (EBSS, 4h) treated RT112 cells. White arrow shows the peripheral clustering of lysosomes. Scale bars equal 10 µm. **B.** Quantification of number of lysosomes per cell in 290 control and 227 rapamycin treated (10 µM for 24h) RT112 cells. ****p<0.0001 in a Mann-Whitney U test, error bars are SEM. **C**. Western blot analysis of siTFEB (72 h, with siRNA pool) in JMSU1 cells and quantification of TFEB levels normalized to GAPDH. Error bars are SEM of 7 independent experiments. **D**. Immunofluorescence staining against the lysosomal-associated membrane protein 1 (LAMP1, CD107a) in JMSU1 cells after TFEB knockdown with individual siTFEB RNAs (72 h). White arrows show the perinuclear clustering of lysosomes. Scale bar is 15 µm. **E**. Western blot of TFEB knockdown with individual siTFEB RNAs (72 h). **F**. Immunofluorescence staining of LAMP1 (red) and protrudin (green) in JMSU1 cells in control (full media) and starvation (EBSS, 24h) treatment conditions. Zoom shows the merged image of the two proteins in the white box. White arrow shows the colocalization between LAMP1 and protrudin. Scale bars are 15 µm. **G**. Quantification of protrudin integrated intensity on lysosomes normalized to total cellular protrudin, in 226 control and 175 starvation (EBSS, 24h) treated RT112 cells; **p<0.01 in a Mann-Whitney U test, error bars are SEM. **H**. Western blot analysis of protrudin in RT112 cells in control (siLUC+DMSO), rapamycin (10 µM for 24h), siTFEB (72h) + rapamycin (10 µM for 24h) and siTFEB (72h) treated conditions. Quantification of protrudin levels and TFEB levels normalized to GAPDH from western blot experiments in RT112 cells, ns p>0.05 in a Kruskal-Wallis test with Dunn's test for multiple comparison. Error bars show SEM. **I**. Western blot analysis of protrudin in RT112 cells in control (full media), rapamycin (10 µM for 24h), starvation (EBSS, 24h) treated conditions and quantification of protrudin levels normalized to GAPDH from western blot experiments, ns p>0.05 in a Kruskal-Wallis test with Dunn's test for multiple comparison. Error bars show SEM. **J**. Western blot analysis of protrudin in JMSU1 cells in control (siLUC) and siTFEB (72 h) conditions and quantification of protrudin normalized to GAPDH from n=3 western blot experiments, ns p>0.05 in a Kruskal-Wallis test with Dunn's test for multiple comparison. Error bars are SEM. **K**. Normalized Log2 RNA expression levels of protrudin (ZFYVE27).

Figure S5:

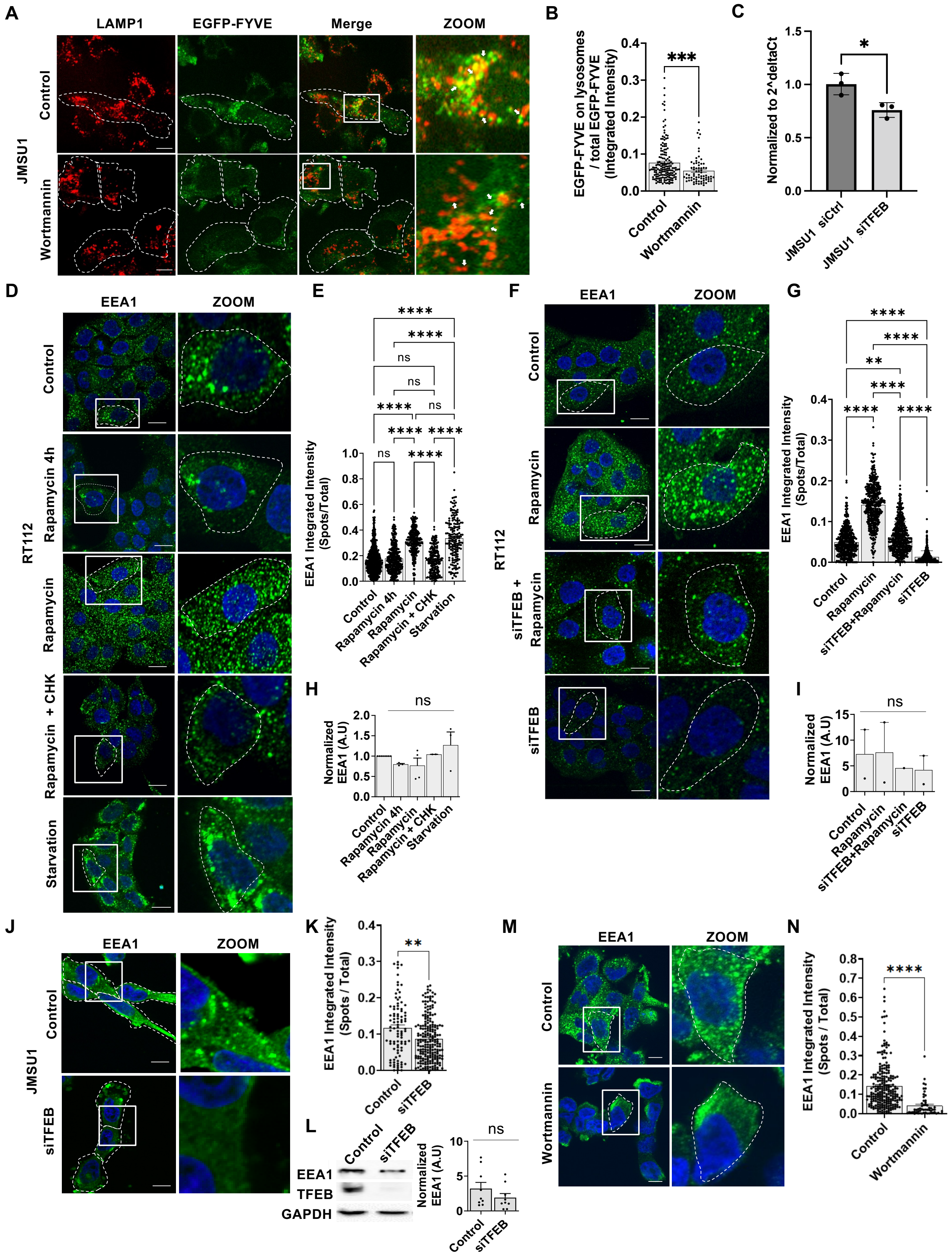
A
LAMP1
EGFP-FYVE
Merge
ZOOM
JMSU1
Control
Wortmannin
B
EGFP-FYVE on lysosomes / total EGFP-FYVE (Integrated Intensity)
***
Control
Wortmannin
C
Normalized to 2^deltaCt
*
JMSU1 siCtrl
JMSU1 siTFEB
D
EEA1
ZOOM
RT112
Control
Rapamycin 4h
Rapamycin
Rapamycin + CHK
Starvation
E
EEA1 Integrated Intensity (Spots/Total)
****
ns
F
EEA1
ZOOM
RT112
Control
Rapamycin
siTFEB + Rapamycin
siTFEB
G
EEA1 Integrated Intensity (Spots/Total)
**
****
siTFEB+Rapamycin
H
Normalized EEA1 (A.U)
ns
I
Normalized EEA1 (A.U)
ns
J
EEA1
ZOOM
JMSU1
Control
siTFEB
K
EEA1 Integrated Intensity (Spots / Total)
**
L
Control
siTFEB
EEA1
TFEB
GAPDH
Normalized EEA1 (A.U)
ns
M
EEA1
ZOOM
Control
Wortmannin
N
EEA1 Integrated Intensity (Spots / Total)
****
Control
Wortmannin

Figure S5. TFEB regulates phosphatidylinositol-3-phosphate levels on endomembranes in bladder cancer cells

A. Immunofluorescence staining of the lysosomal-associated membrane protein 1 (LAMP1/CD107a) (red) in control (DMSO) and wortmannin (1µM, 2h) treated JMSU1 cells transfected with EGFP-FYVE (green). Zoom shows the merged images of LAMP1 and EGFP-FYVE in white box. White arrow shows the colocalization between LAMP1 and EGFP-FYVE. Scale bars equal 10µm. **B**. Quantification of EGFP-FYVE integrated intensity on lysosomes normalized to total cellular EGFP-FYVE, in 165 control and 72 wortmannin (2h) treated JMSU1 cells; *** $p<0.001$ in a Mann-Whitney U test, error bars are SEM. **C**. Quantitative RT-PCR of PIK3C3 expression in JMSU1 cells in control (siLUC) and siTFEB (72 h) treatment conditions normalized to beta actin gene expression from n=3 experiments, * $p<0.05$ in a t-test. Error bars are SD. **D**. Representative images of EEA1 staining in RT112 cells in control (DMSO), rapamycin (10 µM, 24 h) rapamycin (10 µM, 24 h), rapamycin (10 µM, 24 h) + cycloheximide (20 µg/mL, 24 h) and starvation (EBSS, 24h) treated conditions. Zoom shows one single cell in white box. Scale bars are 15 µm. **E**. Quantification of EEA1 integrated intensity on segmented spots normalized to corresponding total cellular EEA1 in 566 control (DMSO), 330 rapamycin (4 h), 245 rapamycin (24 h) , 201 rapamycin (24 h) +cycloheximide (24 h) and 172 starvation (24h) treated RT112 cells; ns $p>0.01$, ****$p<0.0001$, ***$p<0.001$ in a Kruskal-Wallis test with Dunn's test for multiple comparison, error bars are SEM. **F**. Representative images of EEA1 staining in RT112 cells in control (siLUC + DMSO), rapamycin (siLUC + 10 µM, 24 h), siTFEB (72h) + rapamycin (10 µM, 24 h) and siTFEB (72h) treated conditions. Zoom shows one single cell in white box. Scale bars are 15µm. **G.** Quantification of EEA1 integrated intensity on segmented spots normalized to corresponding total cellular EEA1 in 481 control (siLUC), 435 rapamycin (24 h), 481 siTFEB (72h) + rapamycin (24 h) and 626 siTFEB treated RT112 cells; **$p<0.01$, ****$p<0.0001$ in a Kruskal-Wallis test with Dunn's test for multiple comparison, error bars are SEM. **H**. Quantification of western blot of EEA1 normalized to GAPDH in RT112 cells in control (DMSO), rapamycin (4h), rapamycin (24h), rapamycin (24h) + cycloheximide (24h) and starvation (24h) treatment conditions from n=3 experiments. ns $p>0.05$ in a Kruskal-Wallis test with Dunn's test for multiple comparison. Error bars are SEM. **I**. Quantification of western blot of EEA1 normalized to GAPDH in RT112 cells in control (siLUC + DMSO), rapamycin (24h), rapamycin (24h) + siTFEB (72h) and starvation (24h) treatment conditions from n=2 experiments. ns $p>0.05$ in a Kruskal-Wallis test with Dunn's test for multiple comparison. Error bars are SEM. **J**. Representative images of EEA1 staining in JMSU1 cells in control (siLUC) and siTFEB (72 h) conditions. Zoom shows one single cell in white box. Scale bars are 15 µm. **K**. Quantification of EEA1 integrated intensity on segmented spots normalized to corresponding total cellular EEA1, in 94 control (siLUC) and 212 siTFEB treated (72h) JMSU1 cells; ** $p<0.01$ in Mann-Whitney U test. Error bars are SEM. **L**. Western blot analysis and quantification of EEA1 in JMSU1 cells in control (siLUC) and siTFEB (72 h) conditions, error bars are SEM from 9 independent experiments. ns $p>0.05$ in a Mann-Whitney U test. Error bars are SEM. **M.** Immunofluorescence staining of early endosome antigen 1 (EEA1) in control

(DMSO) and wortmannin (1 µM) treated JMSU1 cells. Zoom shows one single cell in white box. Scale bars equal 15 µm. **N**. Quantification of EEA1 integrated intensity on segmented spots normalized to corresponding total cellular EEA1, in 228 control and 56 wortmannin treated JMSU1 cells; **** $p<0.0001$ in a Mann-Whitney U test. Error bars are SEM.

Figure S6: Original, uncropped WB images

Blot images with molecular marker

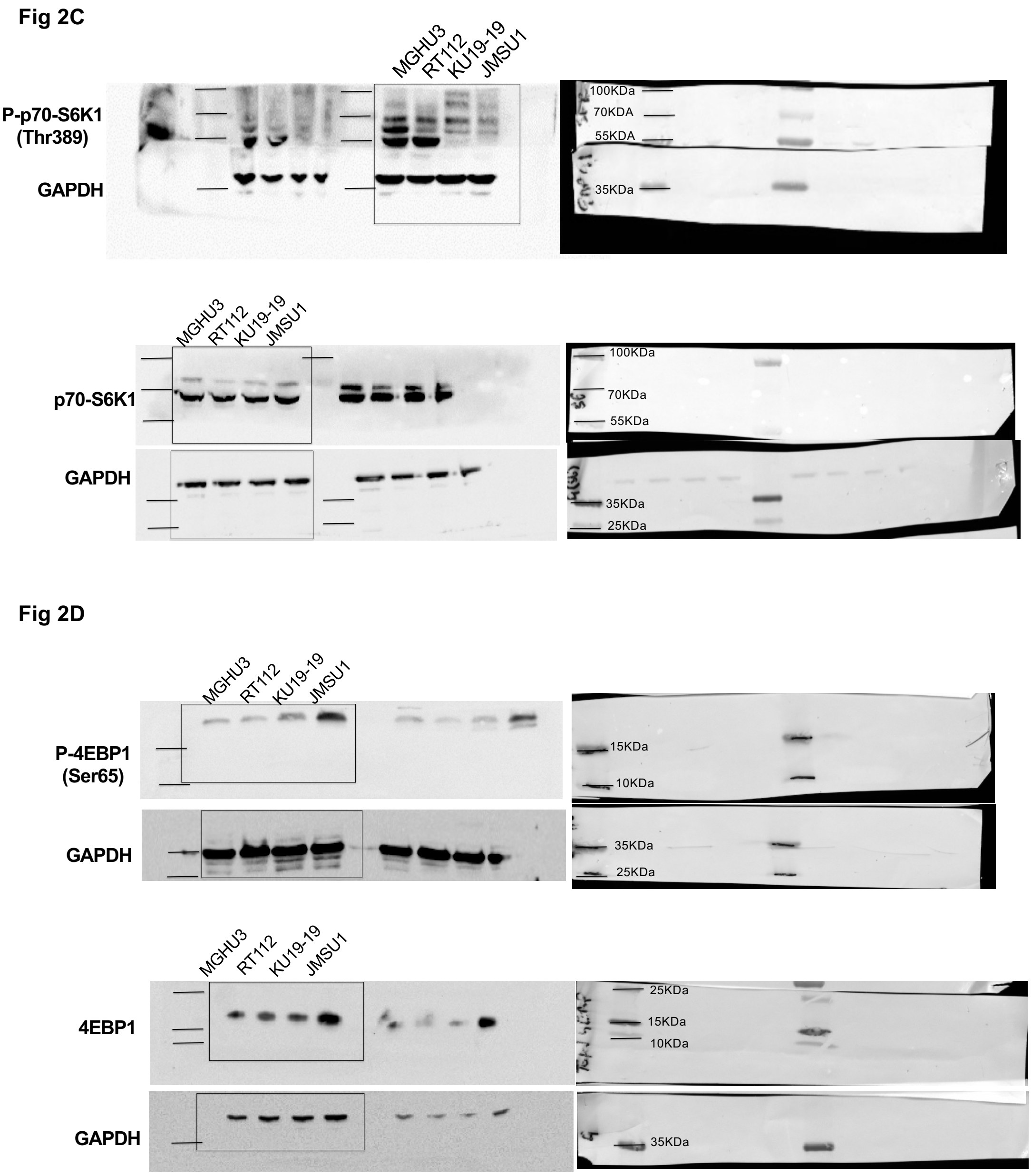

Fig 3C

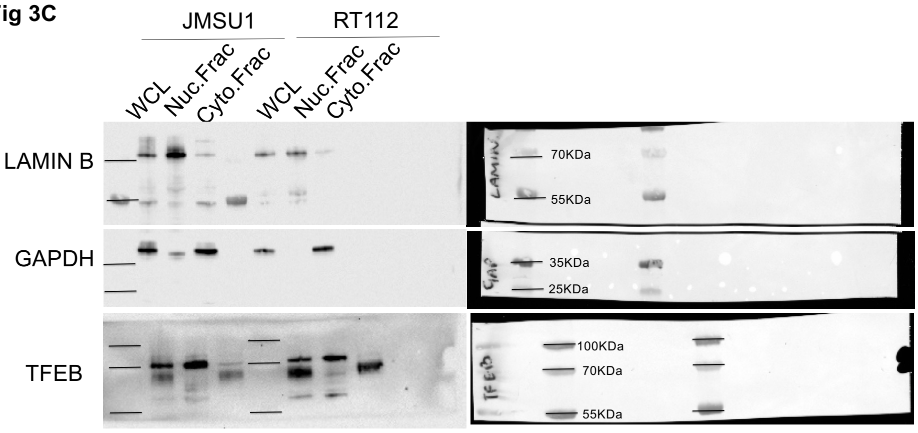
JMSU1
RT112
WCL
Nuc.Frac
Cyto.Frac
WCL
Nuc.Frac
Cyto.Frac
LAMIN B
GAPDH
TFEB
70KDa
55KDa
35KDa
25KDa
100KDa
70KDa
55KDa

Fig 5E

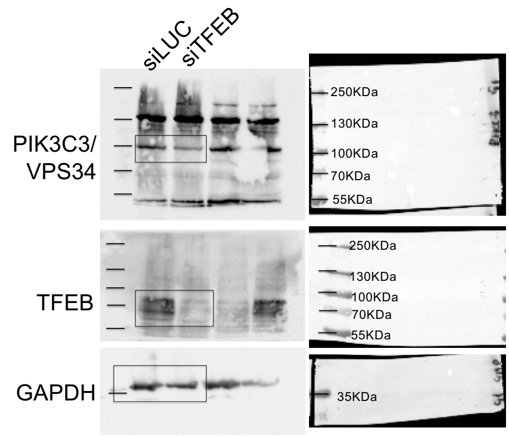
JMSU1
siLUC
siTFEB
PIK3C3/
VPS34
TFEB
GAPDH
250KDa
130KDa
100KDa
70KDa
55KDa
250KDa
130KDa
100KDa
70KDa
55KDa
35KDa

Fig S2C

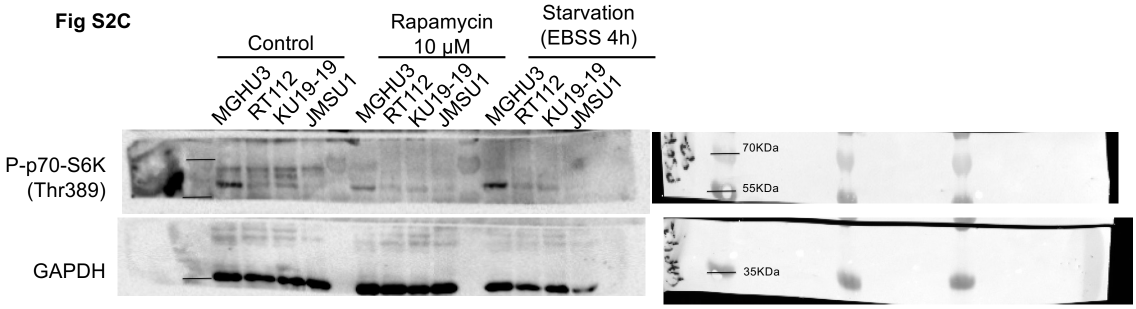

Fig S2D

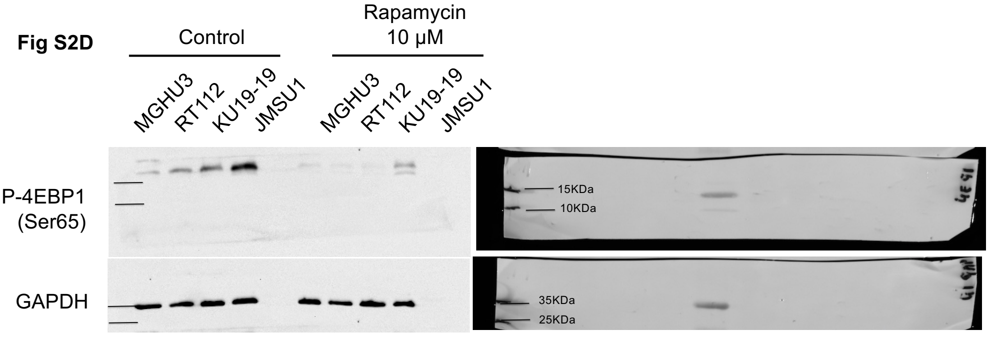

Fig S2E

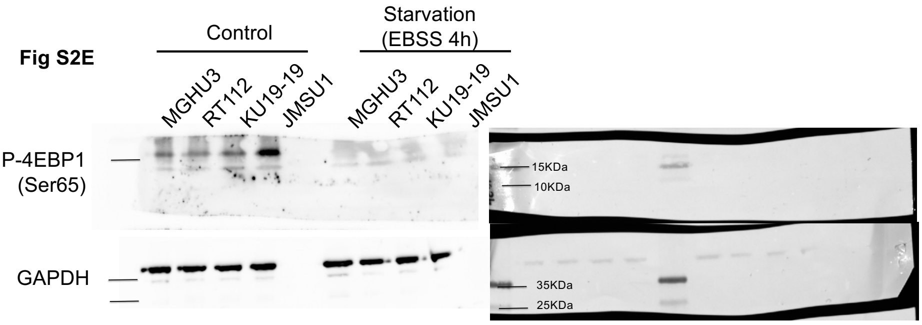

Fig S4C

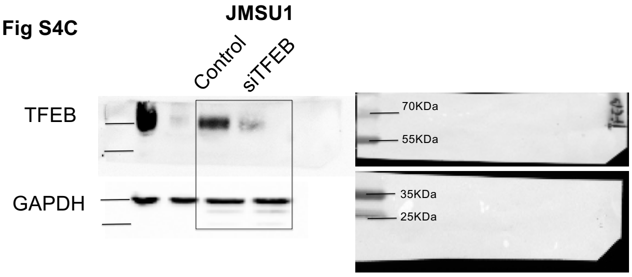

Fig S4E

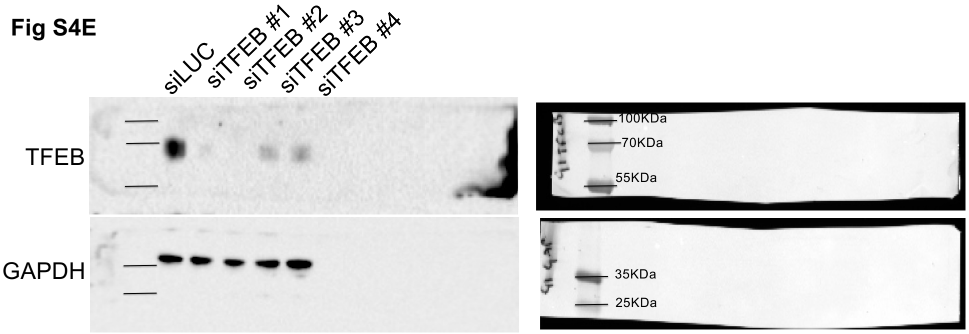

Fig S4H

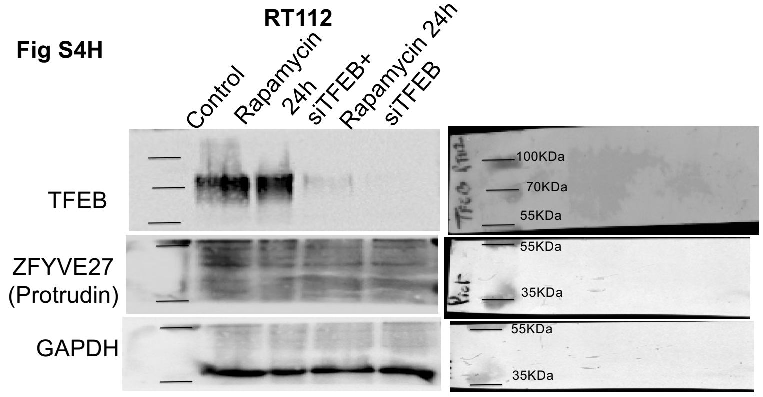

Fig S4I

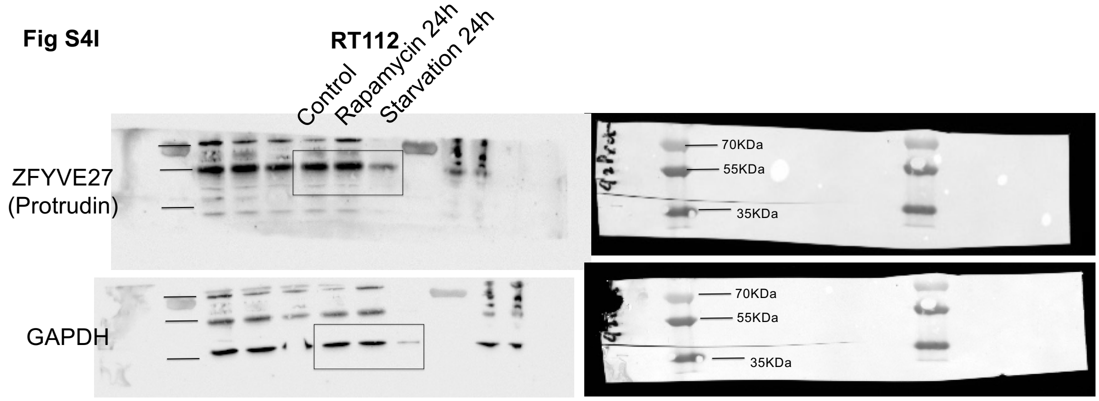

Fig S4J

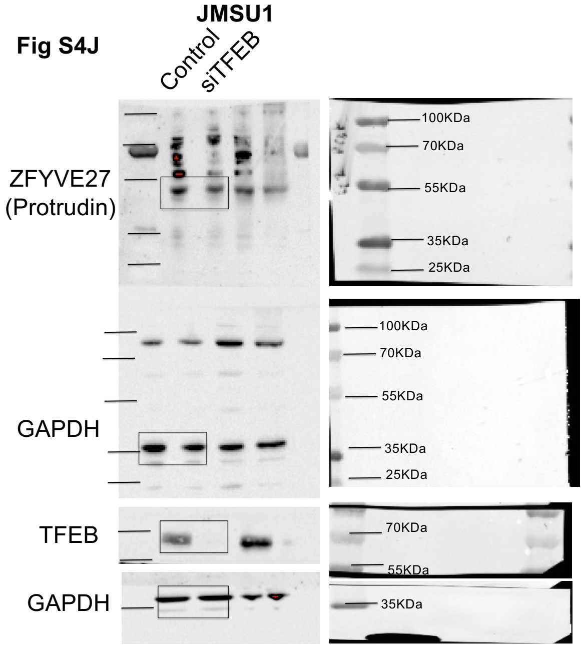

Fig S5L

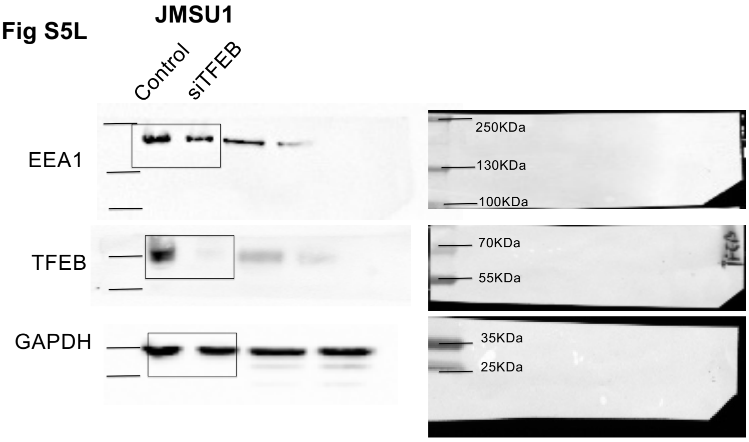
JMSU1
Control
siTFEB
EEA1
TFEB
GAPDH
250KDa
130KDa
100KDa
70KDa
55KDa
35KDa
25KDa
