# Supplementary material for: Transcription factor EB regulates phosphatidylinositol-3-phosphate levels that control lysosome positioning in the bladder cancer model
Source: Commun Biol. 2023 Jan 28;6:114. doi: 10.1038/s42003-023-04501-1 (PMC9884284; doi:10.1038/s42003-023-04501-1)
Supplement: Supplementary file 3 — Description of Additional Supplementary Files [file 42003_2023_4501_MOESM3_ESM.pdf]

Description of Additional Supplementary Files

File name: Supplementary Data 1

Description: The source data behind Fig1 in the paper

Fig.1B NHU: Number of analyzed cell, the index and corresponding coordinates (X,Y in pix and Z in planes) of each lysosome for 76 NHU cells from 3 experiments
Fig.1B RT4: Number of analyzed cell, the index and corresponding coordinates (X,Y in pix and Z in planes) of each lysosome for 73 RT4 cells from 3 experiments
Fig.1B MGHU3: Number of analyzed cell, the index and corresponding coordinates (X,Y in pix and Z in planes) of each lysosome for 65 MGHU3 cells from 3 experiments
Fig.1B RT112: Number of analyzed cell, the index and corresponding coordinates (X,Y in pix and Z in planes) of each lysosome for 64 RT112 cells from 3 experiments
Fig.1B KU19-19: Number of analyzed cell, the index and corresponding coordinates (X,Y in pix and Z in planes) of each lysosome from 64 KU19-19 cells from 3 experiments
Fig.1B T24: Number of analyzed cell, the index and corresponding coordinates (X,Y in pix and Z in planes) of each lysosome from 72 T24 cells from 3 experiments
Fig.1B TCCSup: Number of analyzed cell, the index and corresponding coordinates (X,Y in pix and Z in planes) of each lysosome from 48 TCCSup cells from 2 experiments
Fig.1B JMSU1: Number of analyzed cell, the index and corresponding coordinates (X,Y in pix and Z in planes) of each lysosome from 60 JMSU1 cells from 3 experiments
Fig.1C: Lysosome NND values in µm in cell lines NHU, RT4, MGHU3, RT112, KU19-19, T24, TCCSup, JMSU1
Fig.1D: Average number of lysosomes per cell in cell lines NHU, RT4, MGHU3, RT112, KU19-19, T24, TCCSup, JMSU1
Fig.1E: Average lysosome volume in A.U in cell lines NHU, RT4, MGHU3, RT112, KU19-19, T24, TCCSup, JMSU1

File name: Supplementary Data 2

Description: The source data behind Fig2 in the paper

Fig.2B: Values of mTOR integrated intensity on lysosomes normalized to total mTOR intensity in the cell in cell lines MGHU3, RT112, KU19-19, JMSU1
Fig.2C: P-p70-S6K1 signals normalized to GAPDH in cell lines MGHU3, RT112, KU19-19, JMSU1 derived from multiple experiments
Fig.2D: P-4EBP1 signals normalized to GAPDH in cell lines MGHU3, RT112, KU19-19, JMSU1 derived from multiple experiments

File name: Supplementary Data 3

Description: The source data behind Fig3 in the paper

Fig.3B: Values of % of total mean intensity of TFEB-EGFP in nucleus in the cell lines MGHU3, RT112, KU19-19, JMSU1
Fig.3D: TFEB signals in nuclear fraction (Nuc.Frac) and cytosolic fraction (Cyto.Frac) in cell lines RT112 and JMSU1 from multiple experiments
Fig.3E: Normalized transcriptomic data of 2X TCCSup, 2X T24, 1X JMSU1, 1X KU1919, 1X RT4, 3X MGHU3 and 2X RT112 cells used for the Gene Set Enrichment Analysis (GSEA) of TFEB regulated CLEAR network genes

Fig.3G: Values of % of total mean intensity of TFEB- EGFP in nucleus in RT112 cells in control, rapamycin and rapamycin+MLSI-1 treatment conditions
Fig.3I: Values of % of total mean intensity of TFEB- EGFP in nucleus in JMSU1 cells in control, MLSI-1 and BAPTA treatment conditions

File name: Supplementary Data 4

Description: The source data behind Fig4 in the paper

Fig.4C: Lysosome NND values in µm in RT112 cells in control and rapamycin treatment conditions
Fig.4F: Lysosome NND values in µm in JMSU1 cells in control and siTFEB treatment conditions
Fig.4H: Values of protrudin integrated intensity on lysosomes normalized to total protrudin intensity in RT112 cells in control, rapamycin, siTFEB+ rapamycin and siTFEB treatment conditions
Fig.4J: Values of protrudin integrated intensity on lysosomes normalized to total protrudin intensity in JMSU1 cells in control and siTFEB treatment conditions

File name: Supplementary Data 5

Description: The source data behind Fig5 in the paper

Fig.5B: Values of EGFP-FYVE integrated intensity on lysosomes normalized to total EGFP-FYVE intensity in JMSU1 cells in control and siTFEB treatment conditions
Fig.5D: Values of EGFP-FYVE integrated intensity on endomembranes (spots) normalized to total EGFP-FYVE intensity in JMSU1 cells in control and siTFEB treatment conditions
Fig.5F: PIK3C3/ VPS34 signals normalized to GAPDH in JMSU1 cells from multiple experiments
Fig.5I: Lysosome NND values in µm in JMSU1 cells in control and wortmannin treatment conditions
